# Indirect revascularization vs. non-surgical treatment for Moyamoya disease and Moyamoya syndrome: A comparative effectiveness study

**DOI:** 10.3389/fneur.2022.1041886

**Published:** 2022-12-19

**Authors:** Yixuan Wang, Miao Li, Jie Wang

**Affiliations:** ^1^Department of Neurology, China-Japan Union Hospital, Jilin University, Changchun, Jilin, China; ^2^Department of Neurosurgery, China-Japan Union Hospital, Jilin University, Changchun, Jilin, China

**Keywords:** Moyamoya disease, Moyamoya syndrome, surgical treatment, perioperative complications, quantitative analysis, prognosis

## Abstract

**Background:**

The efficacy of indirect revascularization vs. non-surgical treatment in adults with Moyamoya disease (MMD) and Moyamoya syndrome (MMS) remains controversial.

**Objective:**

To compare the clinical outcomes of indirect revascularization and non-surgical treatments in adult patients with MMD and MMS.

**Methods:**

We collected medical records and follow-up results of adult patients with MMD and MMS who received treatment in the China-Japan Union Hospital of Jilin University between January 2019 and December 2021. A Shapiro–Wilk test, independent sample *t*-test or Mann-Whitney U-test, and Pearson chi-square test were used to compare baseline variables. The propensity-score analysis was used to compare clinical outcomes of patients with MMD and MMS who underwent indirect revascularization and non-surgical treatments. The color-coded digital subtraction angiography (CC-DSA) was used to quantitatively analyzed the preoperative and postoperative (at 6-month follow-up) images of patients in the surgical group.

**Results:**

A total of 144 patients were included in this study, of whom 37 received indirect revascularization treatment and 107 received non-surgical treatment. The average age of the patients was 58.3 ± 13.4 years. Perioperative complications were observed in eight of the operations. During the follow-up period, a total of 35 stroke events occurred, including two cases (5.4%) in the surgery group and 33 cases (30.8%) in the non-surgery group (*p* < 0.05). The preoperative mean transit time (MTT) of bypass vessel (superficial temporal artery, STA) was 0.26 ± 0.07, and the postoperative MTT of bypass vessel was 3.0 ± 0.25, and there was no statistical difference between the subgroups.

**Conclusion:**

Indirect revascularization surgery can significantly reduce the recurrent stroke incidence of MMD and MMS patients.

## Introduction

Moyamoya disease (MMD) is a chronic progressive cerebrovascular disease of unknown etiology. It is characterized by stenosis or occlusion of the terminal portion of the internal carotid artery (ICA), or proximal portions of the anterior and/or the middle cerebral artery, and is accompanied by the formation of abnormal vascular networks at the base of the brain. It is a cerebrovascular disease with high incidence rates in East Asian populations (1, 2). The arteriographic findings are bilateral in typical MMD. However, some patients have only unilateral involvement at the onset of symptoms, and this portion of patients is classified as having Moyamoya syndrome (MMS). Research has demonstrated that as this disease progresses, about 8.3–40% of patients who initially present with unilateral symptoms eventually develop bilateral disease manifestations (3–6). Therefore, the natural history of MMS patients requires investigation, and drug or surgical treatment should be administered at an appropriate time.

The clinical manifestations of patients with MMD and MMS can be divided into two phenotypes, ischemic and hemorrhagic. Although some studies have demonstrated that revascularization of the Moyamoya vascular region can effectively improve the neurocognitive function of patients and reduce the incidence of stroke, postoperative complications during the perioperative period, such as postoperative ischemia, hyperperfusion, impaired wound healing, and subdural effusion, means that the overall benefit of surgical treatment to patients remains controversial (7–10). Therefore, we conducted this retrospective study to investigate the benefit of surgical vs. non-surgical treatment for patients with MMD and MMS by comparing different treatment modalities and their clinical outcomes.

## Materials and methods

### Study design and participants

The participants included in this study were from a cohort of patients diagnosed with MMD and MMS who received treatment in the China-Japan Union Hospital of Jilin University between January 2019 and December 2021. Consecutive cases involving MMD and MMS patients that underwent treatment in the Department of Neurology and Neurosurgery of the China-Japan Union Hospital, Jilin University, were reviewed. Inclusion criteria were as follows: (1) Patients with MMD or MMS identified by digital subtraction angiography (DSA) and/or magnetic resonance angiography (MRA) according to the guidelines for diagnosing and treating MMD developed by the Spontaneous Occlusion of the Circle of Willis Research Committee (2012) (11). (2) Patients over 18 years of age at diagnosis. Exclusion criteria were patients with MMS caused by other definite diseases such as neurofibromatosis type 1, Down's syndrome, and sickle cell disease, among others. This study was conducted in agreement with the Helsinki Declaration and approved by the Research Ethics Committee of the China-Japan Union Hospital, Jilin University. All patients involved in this study gave their informed consent.

### Treatment modalities

Indications for revascularization were determined according to treatment guidelines established by the Spontaneous Occlusion of the Circle of Willis Research Committee (2012) (11). The specific surgical treatment principles are as follows: first, revascularization is the preferred treatment modality for symptomatic affected hemispheres, for both ischemic and hemorrhagic Moyamoya disease. Second, if the patient's symptoms did not significantly improve after surgery on one hemisphere, or if the patient had symptoms in the contralateral hemisphere, a second revascularization could be performed on the contralateral hemisphere. Third, the final decision on whether to proceed with the surgery was made by the neurosurgeon and the patient or the patient's family. It should be noted here that due to objective factors such as economy and culture, some patients and their families refused surgical treatment and chose conservative treatment. Therefore, if the patient or the patient's family decided not to accept surgical procedures, surgery was not performed.

#### Surgical procedures

In this study, indirect bypass surgery was used for all surgeries. Indirect bypass mainly involved encephaloduroarteriosynangiosis (EDAS) and encephalomyosynangiosis (EMS). For EDAS, the scalp artery with the strip of galea was freed from the pericranium and fascia below, a linear dural incision was made, or a strip of dura mater was removed to suture the strip of galea to the dura mater. For EMS, the temporal muscles detached from the bone flap were placed over the cortical surface, and the outer edge of the muscles was sutured to the opened dura mater (12).

#### Conservative treatment

All patients in the non-surgical group were treated with a standardized drug regimen. According to the Chinese expert consensus on treating Moyamoya disease (13), for patients with ischemic MMD and MMS without microbleeds as determined by susceptibility weighted imaging, we used aspirin, clopidogrel, statins, and edaravone for antiplatelets, which improves endothelial function, and neurotrophic therapy. For patients with hemorrhagic or non-specific MMD and MMS, we performed secondary prevention therapy, such as controlling risk factors such as blood pressure, blood sugar, blood lipids, smoking, and alcohol consumption.

### Data collection

We collected disease-related information from the medical records of all enrolled patients, including gender, age, initial symptoms, past medical history, neurological status, cognitive function, and Suzuki stages. Neurological status was evaluated with the modified Rankin Scale (mRS) on admission and at discharge. Cognitive function was evaluated with the MiniMental State Examination (MMSE). The patients were divided into three subgroups according to their initial symptoms: hemorrhage group, ischemia group [cerebral infarction and transient ischemic attack (TIA)], and non-specific symptom group.

For patients in the surgical group, we also need to collect postoperative complications and postoperative Matsushima grades used to evaluate postoperative angiographic outcomes. Postoperative complications, including new infarction, hyperperfusion, impaired wound healing, and subdural effusion, were recorded in detail. New infarction was defined as an acute diffusion-weighted imaging lesion at the time of recurrent clinical stroke. If the patient presented with pulsatile ipsilateral frontotemporal or periorbital pain, diffuse headache, nausea and vomiting, disturbance of consciousness, macular edema or visual disturbance, focal seizures, focal neurological impairment, intracranial or subarachnoid hemorrhage after revascularization, and CT perfusion/MR perfusion confirmed the imaging manifestations of hyperperfusion, the patient could be diagnosed with postoperative cerebral hyperperfusion syndrome (CHS) (14). The Matsushima grading system was proposed by Matsushima in 1992. The criteria of the grading were the proportion of MCA distribution supplied by the surgical revascularization; more than two-thirds of the distribution of MCA is classified as class A, between two-thirds and one-third of the distribution of MCA is classified as class B, and no more than one-third of the distribution of MCA is classified as class C.

### Follow-up

CC-DSA was used to evaluate the collateral vessel formation 6 months after surgery for patients in the surgical group. All patients were followed up *via* telephone interviews or face-to-face visits each year. The information collected at follow-ups includes recurrent strokes (intracranial hemorrhage and cerebral ischemia) and mRS and MMSE scoring.

### Quantitative assessment

In addition, we also quantitatively analyzed preoperative and postoperative (at 6-month follow-up) images of patients in the surgical group using color-coded digital subtraction angiography (CC-DSA). Based on 2D DSA images, CC-DSA created pseudo-color blood flow images through post-processing software, without the need to collect additional image data. For the manually determined region of interest (ROI), CC-DSA could automatically draw a time vs. intensity curve according to the time when the contrast agent concentration in each pixel reaches the peak, and provided the following parameters:

**ROI peak time (s)**: time that contrast intensity of selected ROI reached the peak value in seconds.

**Mean transit time (MTT) (s)**: average contrast agent transit time through the target region.

**Figure 1:**
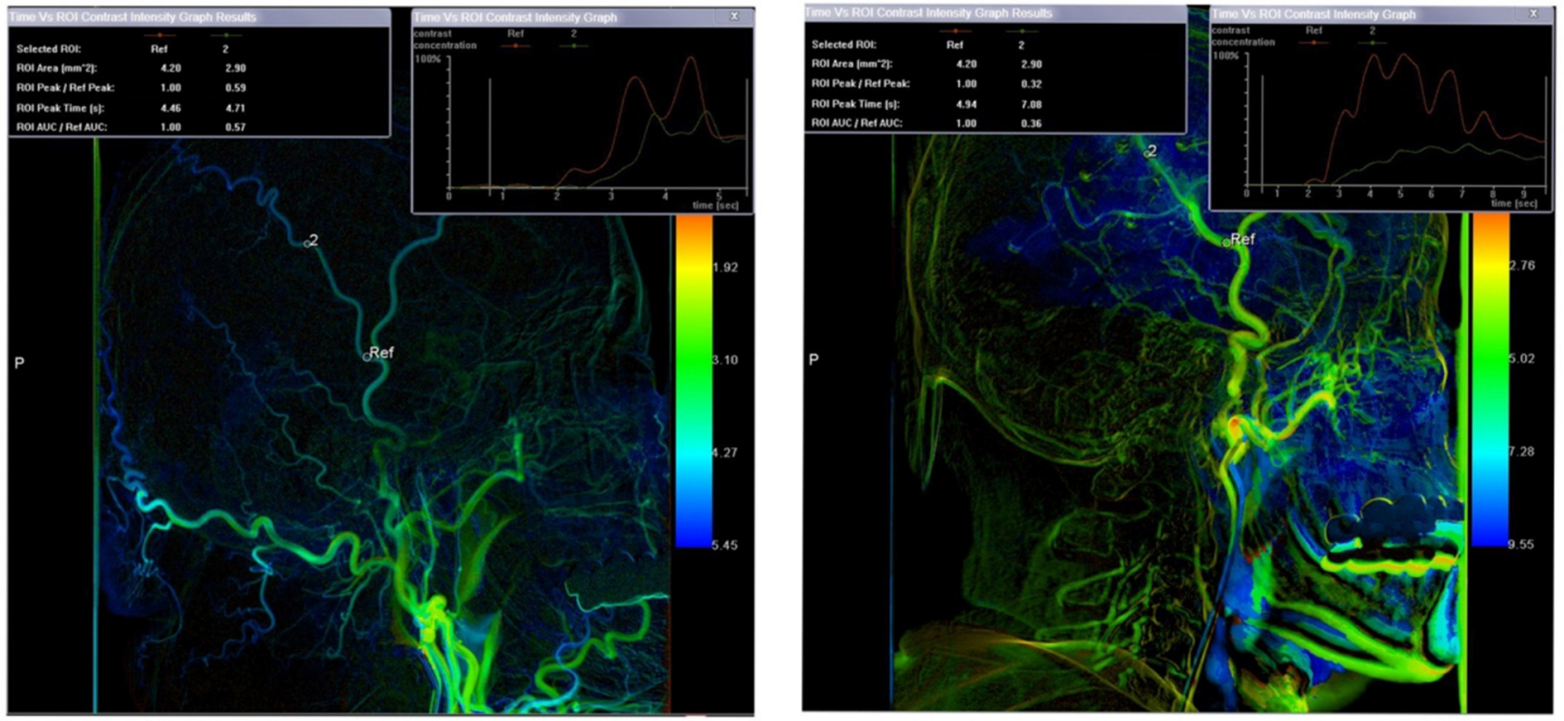
Color-coding digital subtraction angiography (CC-DSA) for Moyamoya disease (MMD) and the measurement principle of hemodynamic parameter. Preoperative view **(left)**, postoperative view **(right)**.

For the bypass vessel (superficial temporal artery, STA), the first ROI was set at the origin of the frontal branch of the STA, and the second ROI was set at the end of the frontal branch of the STA (Figure 1). STA-MTT was defined as the difference time between the two ROI peak times above. We calculated the MTT of changes (ΔMTT) in the superficial temporal artery before and after indirect bypass surgery, and the formula was as follows:


$$\begin{array}{l} \text{ΔSTA − MTT = follow − upSTA − MTT − preoperative STA }\\ \text{ − MTT.} \end{array}$$

The quantitative measurement of CC-DSA was independently conducted by 2 radiologists with more than 5 years of clinical experience in the radiology department of our hospital. A series of training programs were performed in order to ensure the accuracy of data measurement, such as the position of their measurement must be exactly the same before and after surgery, the diameter of the ROI must be exactly the same as the diameter of the blood vessel.

### Statistical analysis

The categorical variables were presented as counts (percentages), and continuous variables were presented as means ± standard deviation. A Shapiro–Wilk test, independent sample *t*-test or Mann-Whitney U-test, and Pearson chi-square test were used to compare variables, as appropriate. The propensity-score analysis was used to compare outcomes in the surgical and non-surgical groups. The aim of this approach was to reduce baseline data bias and confounders in the surgical and non-surgical groups. The age, sex, initial symptoms, past medical history (hypertension and diabetes), admission mRS score, MMSE score, preoperative Suzuki stage, and discharge mRS score of all patients were taken into account, and a propensity score was generated for each measure. We used a 1:1 ratio for matching to obtain a higher degree of case matching and more accurately compare the follow-up results of matched cases. All statistical analyses were performed in SPSS (Version 26.0; IBM, Armonk, New York), and a *p*-value < 0.05 was considered statistically significant.

## Results

### Baseline information

The characteristics and baseline information of the patients are shown in Table 1. A total of 144 patients were included in this study, including 98 males (68.1%) and 46 females (31.9%). The average age of the patients was 58.3 ± 13.4 years. The initial symptoms of the patients were ischemia in 122 cases (84.7%), hemorrhage in 13 cases (9.0%), and non-specific symptoms in nine cases (6.3%). Seventy-three patients (50.7%) had a history of hypertension, and 35 patients (24.3%) had a history of diabetes. The mean admission mRS score for all patients was 2.3 ± 1.2, and the mean admission MMSE score was 15.5 ± 8.7. All patients had Suzuki class III and above.

**Table 1:** Baseline characteristics of all patients.

**Characteristics**		**Treatment modalities**		
	**All patients (*n* = 144)**	**Surgical group (*n* = 37)**	**Non-surgical group (*n* = 107)**	***P*-value**
Age of onset (years)	58.3 ± 13.4	54.8 ± 14.3	59.5 ± 13.0	0.065
Sex
Male	98 (68.1)	24 (64.9)	74 (69.2)	0.629
Female	46 (31.9)	13 (35.1)	33 (30.8)	
Initial symptoms
Ischemic	122 (84.7)	33 (89.2)	89 (83.2)	0.273
Infarction	115 (79.9)	30 (81.1)	85 (79.4)	
TIA	7 (4.9)	3 (8.1)	4 (3.7)	
Hemorrhagic	13 (9.0)	1 (2.7)	12 (11.2)	
Non-specific	9 (6.3)	3 (8.1)	6 (5.6)	
Headache	3 (2.1)	1 (2.7)	2 (1.9)	
Epilepsy	5 (3.5)	2 (5.4)	3 (2.8)	
Hemichorea	1 (0.7)	0 (0.0)	1 (0.9)	
Past medical history
Hypertension	73 (50.7)	18 (48.6)	55 (51.4)	0.998
Diabetes	35 (24.3)	9 (24.3)	26 (24.3)	0.773
Admission mRS score
Mean	2.3 ± 1.2	1.8 ± 1.0	2.5 ± 1.2	0.001
0–2	86 (59.7)	29 (78.4)	57 (53.3)	0.001
3–6	58 (40.3)	8 (21.6)	50 (46.7)	
Admission MMSE score
Mean	15.5 ± 8.7	14.2 ± 7.5	15.9 ± 9.0	0.269
0–9	38 (26.4)	11 (29.7)	27 (25.2)	0.337
10–20	60 (41.7)	17 (45.9)	43 (40.2)	
21–26	25 (17.4)	7 (18.9)	18 (16.8)	
27–30	21 (14.6)	2 (5.4)	19 (17.8)	
Preoperative Suzuki stage
Mean	4.2 ± 0.8	4.7 ± 0.9	4.1 ± 0.6	0.000
1	0 (0.0)	0 (0.0)	0 (0.0)	0.000
2	0 (0.0)	0 (0.0)	0 (0.0)	
3	13 (9.0)	1 (2.7)	12 (11.2)	
4	103 (71.5)	19 (51.4)	84 (78.5)	
5	12 (8.3)	8 (21.6)	4 (3.7)	
6	16 (11.1)	9 (24.3)	7 (6.5)	
Preoperative STA-MTT	N/A	0.26 ± 0.07	N/A	
Discharge mRS score
Mean	2.1 ± 1.0	1.5 ± 0.8	2.4 ± 1.0	0.000
0–2	92 (63.9)	33 (89.2)	59 (55.1)	0.000
3–6	52 (36.1)	4 (10.8)	48 (44.9)	

TIA, transient ischemic attack; mRS, modified Rankin Scale; MMSE, MiniMental State Examination; ICA, internal carotid artery; MTT, mean transit time; STA, superficial temporal artery. Values are numbers of cases (%) unless otherwise indicated. Mean values are presented with SDs.

### Postoperative complications of the patients in surgical group

Differentiated by treatment modality, there were 37 patients in the surgery group and 107 patients in the non-surgical group. Revascularization surgery was performed on 37 patients; one male patient received a second operation on the contralateral side, for a total of 38 procedures. Perioperative complications were observed in eight of the operations, including infarction in two (5.3%) cases, subdural effusion in two (5.3%), venous thrombosis in one (2.6%), and pneumonia in three (7.9%). Stratified by symptoms, the incidence of perioperative complications in each group is as follows: six for the ischemic group, zero for the hemorrhagic group, and two for the non-specific group (*p* = 0.119) (Table 2).

**Table 2:** Angiographic outcomes, neurological status and cognitive function of patients in the surgical group.

	**All hemispheres (*n* = 38)**	**Ischemic (*n* = 34)**	**Hemorrhagic (*n* = 1)**	**Non-specific (*n* = 3)**	***P*-value**
Perioperative complications	8 (21.1)	6 (17.6)	0 (0.0)	2 (66.6)	0.119
Infarction	2 (5.3)	1 (2.9)	0 (0.0)	1 (33.3)	0.149
Subdural effusion	2 (5.3)	2 (5.9)	0 (0.0)	0 (0.0)	
Venous thrombosis	1 (2.6)	0 (0.0)	0 (0.0)	1 (33.3)	
Pneumonia	3 (7.9)	3 (8.9)	0 (0.0)	0 (0.0)	
Matsushima grading system
Grade A	15 (39.5)	13 (38.2)	0 (0.0)	2 (66.7)	0.486
Grade B	13 (34.2)	11 (32.4)	1 (100)	1 (33.3)	
Grade C	10 (26.3)	10 (29.4)	0 (0.0)	0 (0.0)	
Follow-up mRS score (at 6 months)
Mean	0.9 ± 1.5	0.9 ± 1.5	0.0 ± 0.0	1.3 ± 2.3	0.713
0–2	32	29	1	2	0.634
3–6	6	5	0	1	
Follow-up MMSE score (at 6 months)
Mean	16.0 ± 7.2	16.3 ± 7.0	15.0 ± 0.0	13.0 ± 11.4	0.458
0–9	7	5	0	2	0.321
10–20	19	18	1	0	
21–26	9	8	0	1	
27–30	3	3	0	0	
Follow-up STA-MTT (s)	3.0 ± 0.25	3.0 ± 0.26	2.98 ± 0.00	2.96 ± 0.19	0.899
ΔSTA-MTT (s)	2.75 ± 0.28	2.76 ± 0.29	2.70 ± 0.00	2.72 ± 0.11	0.509

mRS, modified Rankin Scale; MMSE, MiniMental State Examination; ICA, internal carotid artery; MTT, mean transit time; STA, superficial temporal artery.

### Clinical outcomes

**Figure 2:**
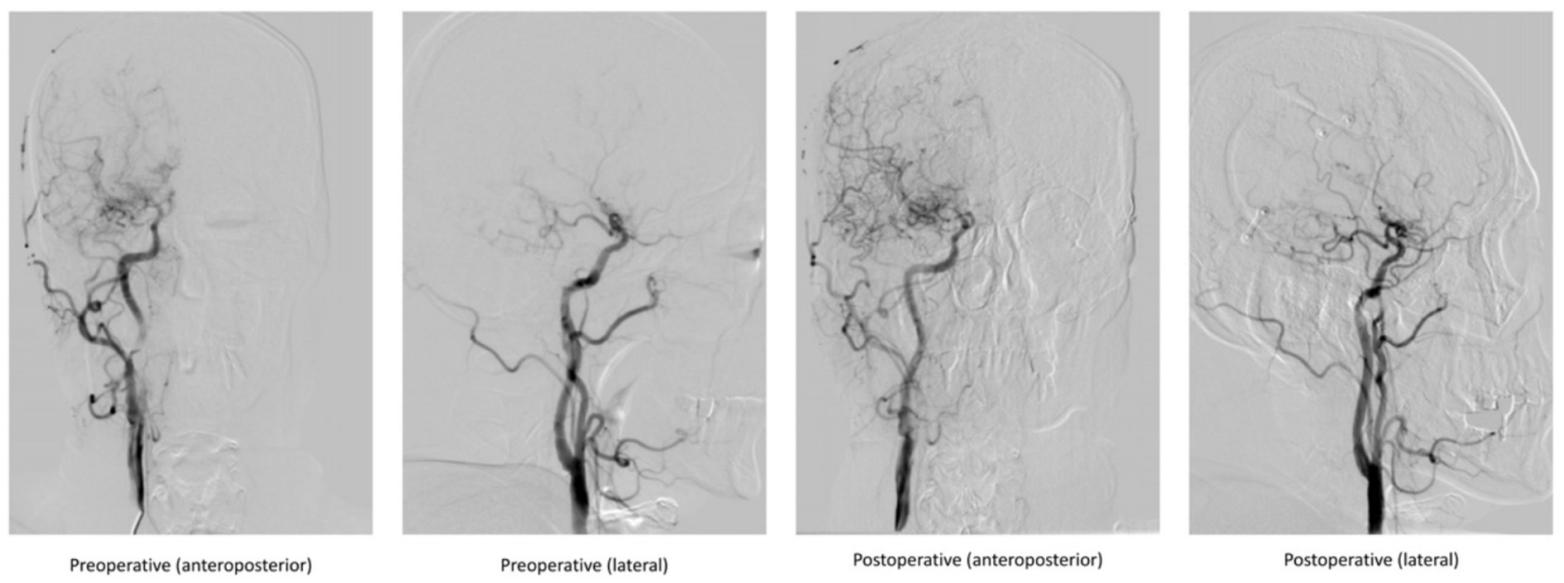
Comparison of DSA images before and after indirect bypass surgery in a 48-year-old male patient.

**Figure 3:**
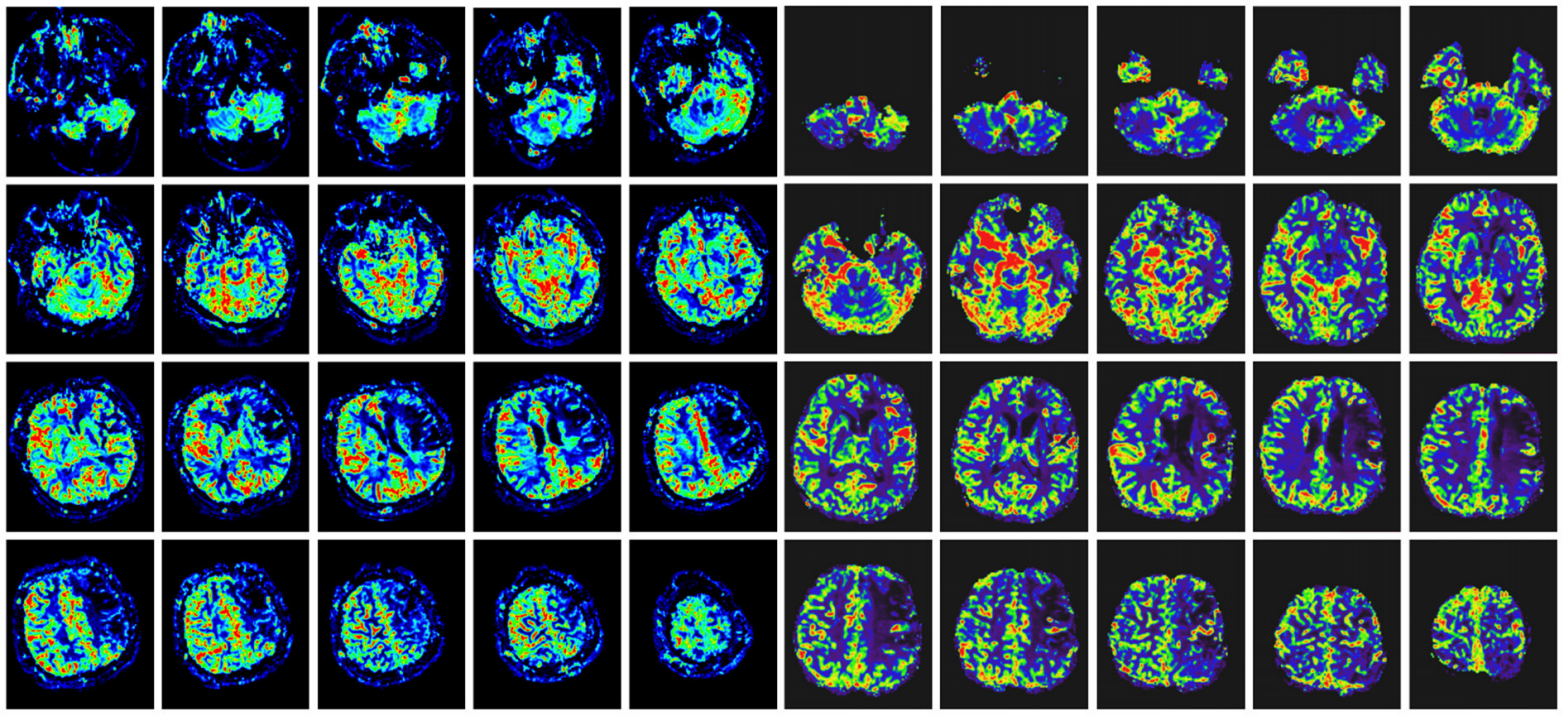
Comparison of head MRI perfusion before surgery **(left)** and after surgery **(right)** in the same patient with Figure 2. The preoperative ischemic region had improved perfusion in the same region after indirect bypass surgery.

**Figure 4:**
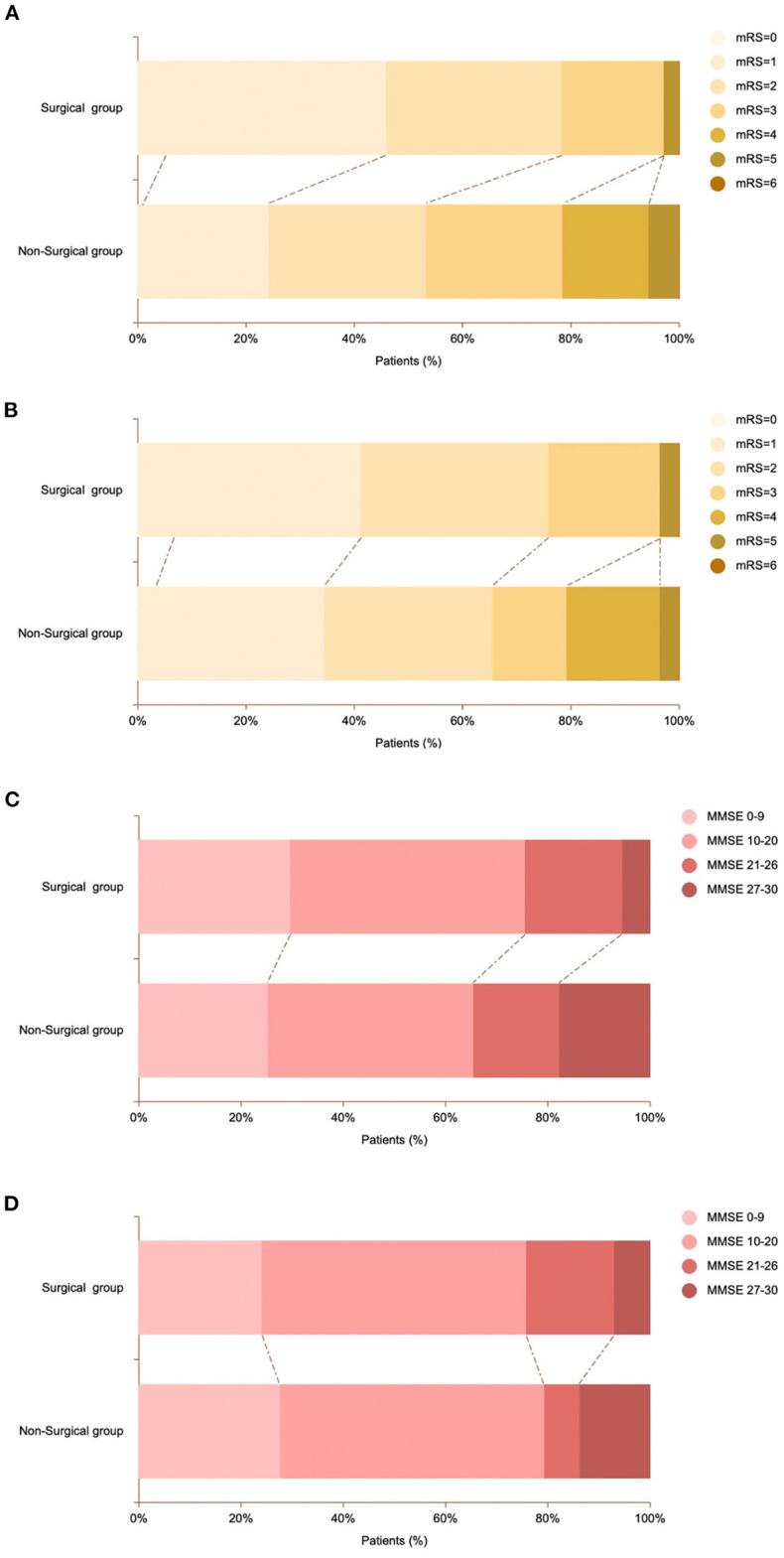
Comparison of mRS scores and MMSE scores of patients in surgical and non-surgical group. **(A)** Proportion of mRS scores ranging from 0 (light yellow) to 6 (brown) at admission for patients in the surgical and non-surgical groups. **(B)** Proportion of mRS scores at admission for surgical and non-surgical patients in propensity score-matched groups. **(C)** Proportion of MMSE scores ranging from 0 (pink) to 6 (dark red) at admission for patients in the surgical and non-surgical groups. **(D)** Proportion of MMSE scores at admission for surgical and non-surgical patients in propensity score-matched groups.

We collected the DSA outcomes of 37 patients in the surgery group 6 months after surgery, as well as the recurrent stroke events, mRS scores, and MMSE scores of all patients (Tables 2, 3). The comparison images of the patient before and after surgery are shown in Figures 2, 3. The mean follow-up time for all patients was 15.2 ± 9.5 months. During the follow-up period, a total of 35 stroke events occurred, including two cases (5.4%) in the surgery group and 33 cases (30.8%) in the non-surgery group (*p* < 0.05). Next, we matched 29 patients in the surgery group with 29 patients in the non-surgery group by propensity score analysis. Baseline information and follow-up information for all patients after propensity score analysis matching are shown in Tables 4, 5. There were no significant differences in age, sex, initial symptom, past medical history, admission mRS score (Figure 4), MMSE score, preoperative Suzuki stage, and discharge mRS score of all patients from matched groups. Data from the matched group showed that patients in the non-surgical group had a much higher rate of recurrent stroke compared with those in the surgical group (86.2 vs. 6.9%, respectively). At the last follow-up, patients in the surgical had a better neurological status (lower mRS score) than at admission; however, patients in the non-surgical group had a slightly worse neurological status than on admission. The performance of cognitive function and neurological status of the patients in the surgery group and the non-surgery group were consistent, with a higher MMSE score at the last follow-up in the surgery group than at admission and a lower MMSE score in the non-surgical group than at admission.

**Table 3:** Neurological status and cognitive function during follow-up in surgical and non-surgical group.

		**Treatment Modalities**		***P*-value**
	**All patients (*n* = 144)**	**Surgical group (*n* = 37)**	**Non-surgical group (*n* = 107)**	
Follow-up (month)	15.2 ± 9.5	18.6 ± 9.3	14.0 ± 9.3	0.002
Recurrent strokes	35 (24.3)	2 (5.4)	33 (30.8)	0.002
Follow-up mRS score
Mean	2.3 ± 1.6	0.9 ± 1.5	2.8 ± 1.4	0.000
0–2	81 (56.3)	31 (83.8)	50 (46.7)	0.000
3–6	63 (43.8)	6 (16.2)	57 (53.3)	
Follow-up MMSE score
Mean	13.9 ± 8.5	16.1 ± 7.3	13.1 ± 8.8	0.092
0–9	45 (31.3)	7 (18.9)	38 (35.5)	0.312
10–20	60 (41.7)	18 (48.6)	42 (39.3)	
21–26	30 (20.8)	9 (24.3)	21 (19.6)	
27–30	9 (6.3)	3 (8.1)	6 (5.6)	

mRS, modified Rankin Scale; MMSE, MiniMental State Examination.

**Table 4:** Comparison of patient characteristics in the surgical and non-surgical groups after propensity-score matching.

**Characteristics**		**Treatment modalities**		
	**All patients (*n* = 58)**	**Surgical group (*n* = 29)**	**Non-surgical group (*n* = 29)**	***P*-value**
Age of onset (years)	56.1 ± 14.1	54.6 ± 14.2	57.7 ± 14.0	0.405
Sex
Male	34 (58.6)	19 (65.6)	15 (51.7)	0.286
Female	24 (41.4)	10 (34.5)	14 (48.3)	
Initial symptoms
Ischemic	49 (84.5)	26 (89.7)	23 (79.3)	0.553
Infarction	46(79.3)	24 (82.8)	22 (75.9)	
TIA	3 (5.2)	2 (6.9)	1 (3.4)	
Hemorrhagic	3 (5.2)	1 (3.4)	2 (6.9)	
Non-specific	6 (10.3)	2 (6.9)	4 (13.8)	
Headache	3 (5.2)	1 (3.4)	2 (6.9)	
Epilepsy	2 (3.4)	1 (3.4)	1 (3.4)	
Hemichorea	1 (1.7)	0 (0.0)	1 (3.4)	
Past medical history
Hypertension	34 (58.6)	17 (58.6)	17 (58.6)	1.000
Diabetes	14 (24.1)	8 (27.6)	6 (20.7)	0.539
Admission mRS score	2.0 ± 1.2	1.8 ± 1.1	2.2 ± 1.3	0.293
0–2	41 (70.7)	22 (75.9)	19 (65.5)	0.387
3–6	17 (29.3)	7 (24.1)	10 (34.5)	
Admission MMSE score	14.7 ± 7.7	14.9 ± 7.4	15.5 ± 8.2	0.828
0–9	15 (25.9)	7(24.1)	8 (27.6)	0.568
10–20	30 (51.7)	15 (51.7)	15 (51.7)	
21–26	7 (12.1)	5 (17.2)	2 (6.9)	
27–30	6 (10.3)	2 (6.9)	4 (13.8)	
Preoperative Suzuki stage	4.5 ± 0.9	4.4 ± 0.8	4.5 ± 0.9	0.949
1	0 (0.0)	0 (0.0)	0 (0.0)	0.482
2	0 (0.0)	0 (0.0)	0 (0.0)	
3	3 (5.2)	1 (3.4)	2 (6.9)	
4	37 (63.8)	19 (65.5)	18 (62.1)	
5	7 (12.1)	5 (17.2)	2 (6.9)	
6	11 (19.0)	4 (13.8)	7 (24.1)	
Preoperative STA-MTT	N/A	0.25 ± 0.07	N/A	
Discharge mRS score	1.1 ± 1.1	0.9 ± 1.0	1.2 ± 1.2	0.319
0–2	51 (87.9)	28 (96.6)	23 (79.3)	0.054
3–6	7 (12.1)	1 (3.4)	6 (20.7)	

mRS, modified Rankin Scale; MMSE, MiniMental State Examination; ICA, internal carotid artery; MTT, mean transit time; STA, superficial temporal artery.

**Table 5:** Neurological status and cognitive function during follow-up in all patients after propensity-score matching.

		**Treatment modalities**		***P*-value**
	**All patients (*n* = 58)**	**Surgical group (*n* = 29)**	**Non-surgical group (*n* = 29)**	
Follow-up (month)	18.7 ± 9.7	20.2 ± 9.5	17.1 ± 9.8	0.091
Recurrent strokes	27 (46.6)	2 (6.9)	25 (86.2)	0.000
Follow-up mRS score	1.9 ± 1.9	1.0 ± 1.6	2.9 ± 1.7	0.000
0–2	41 (70.7)	24 (82.8)	17 (58.6)	0.043
3–6	17 (29.3)	5 (17.2)	12 (41.4)	
Follow-up MMSE score	14.2 ± 7.9	16.8 ± 7.1	11.6 ± 8.0	0.012
0–9	16 (27.6)	4 (13.8)	12 (41.4)	0.048
10–20	28 (48.3)	15 (51.7)	13 (44.8)	
21–26	9 (15.5)	7 (24.1)	2 (6.9)	
27–30	5 (8.6)	3 (10.3)	2 (6.9)	

mRS, modified Rankin Scale; MMSE, MiniMental State Examination.

### Quantitative analysis

The mean preoperative STA-MTT was 0.26 ± 0.07 for all patients in surgical groups, the mean postoperative STA-MTT was 3.0 ± 0.25, and the mean ΔSTA-MTT was 2.75 ± 0.28. The postoperative STA-MTT and ΔSTA-MTT of patients in ischemia group, hemorrhage group and non-specific symptom group were shown in Table 2. There was no statistical difference in postoperative STA-MTT and ΔSTA–MTT among the groups of patients.

## Discussion

Although some studies have shown that revascularization can reduce future stroke events in patients with MMD and MMS (7, 15), few randomized controlled trials have compared the effects of surgical vs. non-surgical treatment in these patients. The natural history and postoperative survival of patients with MMD and MMS remain unclear. This study compared the clinical outcomes of MMD and MMS patients by collecting follow-up results after surgical and non-surgical modalities. Our findings suggest that prompt surgical treatment could be a better option for adults with MMD and MMS. The postoperative neurological status and cognitive function of patients in the surgical group were significantly better than those in the non-surgery group. In patients with either hemorrhagic or ischemic type, the incidence of recurrent stroke after revascularization was significantly lower than in patients who did not undergo revascularization.

A study that documented the long-term natural history of patients with hemorrhagic MMD showed that the annual rebleeding rate in patients with hemorrhagic MMD was 7.09/person/year, and the time between the occurrence of rebleeding and the occurrence of the first intracerebral hemorrhage could be 10 years or longer (16). An observational study of 176 patients with hemorrhagic MMD by Kim et al. found that the overall rebleeding rate was 16.9%/person (95% confidence interval, 11.3–24.8%) 5 years after the initial symptom of hemorrhage and reached 26.3%/person (95% confidence interval, 18.5–36.4%) 10 years after the initial symptoms of hemorrhage (17). Liu et al. summarized and compared the clinical characteristics of ischemic MMD in children and adults and found that compared with children, adult patients are less likely to develop leptomeningeal collaterals, and poor leptomeningeal collaterals are strongly associated with severe clinical symptoms and poor postoperative outcomes (18). In general, the occurrence of recurrent hemorrhagic or ischemic events can further impair the patient's neurological status and cognitive function. Therefore, timely treatment is essential for MMD patients. However, since the situation of MMD patients cannot be generalized, choosing an appropriate treatment modality is the most critical step of treatment.

There is currently no treatment that can prevent and reverse MMD or MMS. Clinically, doctors often use antiplatelet drugs, anti-epileptic drugs, or drugs to relieve headaches to control common symptoms in MMD patients. According to the treatment guidelines established by the Spontaneous Occlusion of the Circle of Willis Research Committee (2012), drug therapy can also be used for patients with acute cerebral infarction and mild MMD. For patients with ischemic MMD, aspirin or clopidogrel is the recommended treatment for preventing long-term recurrent ischemic attacks, despite the lack of corresponding clinical evidence (11). However, the single treatment of a certain drug obviously does not slow MMD progress. A multicenter prospective randomized controlled clinical study of patients with hemorrhagic MMD in Japan showed that cerebrovascular reconstruction surgery could reduce the 5-year rebleeding rate from 31.6 to 11.9% (15). The experience of a single-center study in China, including 528 patients, showed that surgical revascularization could improve cerebral perfusion and positively impact the prevention of rebleeding in patients with hemorrhagic MMD. Cerebrovascular revascularization seems to be a good option for patients with hemorrhagic MMD. In this study, there were significant differences between the surgical and non-surgical groups in the incidence of recurrent stroke events during follow-up and the mRS score at follow-up (both *p* < 0.05). The incidence of recurrent stroke in the non-surgical group was much higher than in the surgery group (30.8 vs. 5.4%). In the subgroup analysis of the surgery group, the neurological status and cognitive function of the ischemia group, hemorrhage group, and non-specific symptom group were not statistically different (both *p* > 0.05), which could indicate that the initial symptoms do not significantly impact the clinical outcomes of MMD and MMS patients. However, postoperative complications in the patients of the surgical group cannot be ignored. A total of eight patients (21.1%) experienced postoperative complications, which was slightly higher than in previous studies (10, 19, 20). However, it should be noted that the average age of the included cases in the study on postoperative complications in patients with MMD conducted by Zhao, Araki, and Hara was <40 years old (10, 19, 20), while the mean age of onset of the patients included in this study was significantly higher than the mean age of patients included in previous studies. This could be why the incidence of age-related postoperative complications, including subdural effusion and pneumonia in this study, is higher than the incidence of postoperative complications in patients with MMD in the published literature. In the region where the author lives, some young patients may show no symptoms and can only be detected by occasional physical examination. However, the elderly patients often presented with ischemic symptoms as the first symptom, which accounted for 84.7% of the patients included in this study. This may be the reason why the average age of patients included in this study was higher than that of patients included in previous literature. In addition, the research center of the author is located in the northeast of China, which is a relatively underdeveloped area, which may also be one of the reasons affecting the age of patients at diagnosis. Another issue is that cases in this study included patients with MMD and MMS, and since the two diseases are roughly the same in terms of clinical manifestations and treatment, subgroup analyses were not separately performed for the two diseases. However, as mentioned earlier, patients with an initial presentation of unilateral Moyamoya vessels have a high probability of progressing to bilateral disease. Therefore, effective intervention at the early stage of symptomatic onset could improve clinical outcomes for patients.

In the quantitative analysis of the postoperative outcomes of the patients in the surgical group, the mean transit time of the bypassed vessels was significantly prolonged after the operation, which means that the collateral circulation of the bypass vessels increased after the surgery, and the blood flow of the corresponding supply area was significantly increased compared with that before the surgery. Since all the patients included in this study underwent indirect bypass surgery, we did not see a direct increase in the blood flow of the MCA in the patients after surgery, however, the quantitative analysis of the bypassed vessels directly showed an improvement in the ischemic area, thus proving that our procedure was reasonable and effective.

In the surgical treatment of MMD, the revascularization method has always been controversial. Kim et al. (21) conducted a meta-analysis of the effects of direct and indirect bypass surgery on symptomatic and hemodynamically unstable adult MMD patients in 2016, including 536 patients and 732 treated hemispheres. Evidence gathered in this study shows that direct bypass or combined bypass surgery is more beneficial for postoperative revascularization in adult patients with MMD. However, the three studies included in this article all mentioned that indirect bypass surgery was equally effective when it was not technically available. Khan et al. (22) recently published a study of 162 patients with MMD, MMS, and steno-occlusive disease treated by direct bypass surgery, which also showed that direct bypass surgery helped to improve neurological function in patients with MMD and MMS. But it's important to note that this study also had a high rate of bypass occlusion (10%). Another meta-analysis noted that although both direct bypass and combined bypass were superior to indirect bypass in the treatment of early stroke, late stroke and late intracerebral hemorrhage, indirect bypass was significantly superior to both direct and combined bypass in the treatment of early intracerebral hemorrhage (23). Sam et al. (24) evaluated the improvement of cerebral blood flow in MMD and steno-occlusive disease patients before and after cerebrovascular revascularization by blood-oxygen-level-dependent cerebrovascular reactivity MRI and computerized prospective targeting of CO_2_, and they found that unilateral revascularization not only reversed gray matter thinning and cognitive loss in the affected hemisphere but also significantly improved in the unaffected hemisphere, however, this study included both direct and indirect bypass surgery, so it cannot be directly stated which surgery method is more effective. In our study, all patients underwent indirect bypass surgery. Compared with direct bypass, indirect bypass operation is relatively simple, can avoid CHS caused by sudden increase in blood flow, and can effectively reduce the incidence of perioperative complications in patients, thereby reducing possible prolonged hospital days and unnecessary hospitalizations cost. It should be noted that this study is a retrospective study, we cannot avoid certain biases, such as the experience of neurosurgeons and the influence of subjective factors on patients. Therefore, studies with larger samples are required to better investigate this issue.

## Conclusions

The clinical outcomes of MMD and MMS patients who received non-surgical treatment are poor, and revascularization surgery can significantly reduce the recurrent stroke incidence of MMD and MMS patients.

## Data availability statement

The original contributions presented in the study are included in the article/supplementary material, further inquiries can be directed to the corresponding author.

## Ethics statement

The studies involving human participants were reviewed and approved by the Research Ethics Committee of the China-Japan Union Hospital, Jilin University. Written informed consent for participation was not required for this study in accordance with the national legislation and the institutional requirements.

## Author contributions

YW: investigation and writing—original draft. YW, ML, and JW: methodology. ML: supervision. JW: validation. All authors contributed to the article and approved the submitted version.
